# Supplementary material for: Disrupted Top‐Down Modulation as a Mechanism of Impaired Multisensory Processing in Autistic Children
Source: Adv Sci (Weinh). 2026 Oct 28:e78528. Online ahead of print. doi: 10.1002/advs.78528 (PMC13715921; doi:10.1002/advs.78528)
Supplement: Supplementary file 1 [file ADVS-9999-e78528-s001.docx]

**Supplementary Figure 1. Absence of statistical differences of race model violation between groups.**

**Supplementary Figure 2. Effect of eye-tracking filtration on ERPs.**

**Supplementary Figure 3. Effect of the baseline correction on ERPs and MSI.** Here we repeated the analysis for unisensory and MSI using a longer baseline time-window: -200 to 0ms (compared to -50 to 20ms in the manuscript). (**A**) Averaged visual and (**B**) auditory ERPs for a cluster of occipital channels for visual, and a cluster of temporal channels for auditory, in NA (green), AU (orange) and SIB (purple). The shaded areas indicate the standard error of the mean (SEM) in the corresponding color. While the visual N1 and auditory N1/P2 were not different between groups, the visual P1 was significantly different between groups (F = 4.49, p = 0.014), post hoc comparisons (Tukey tests) revealed no significant differences between the NA and AU groups (t = -1.40, p = 0.345) or between the AU and SIB groups (t = 1.18, p = 0.470); only the NA and SIB groups differed significantly (t = -2.40, p = 0.047). (**C**) Spatio-temporal cluster plot of T-values (one-sample test against 0) for the difference (AV – [A+V]), with significant clusters outlined in black. Plots are shown separately for NA (left, n = 36), AU (middle, n = 40), and SIB (right, n = 26) groups. (**D**) Grand-average ERPs over a cluster of left parieto-central electrodes (selected from the significant spatial cluster of the permutation statistics for the NA group) in response to audiovisual stimuli (blue), the sum of unisensory stimuli (orange), and their difference (green).

**Supplementary Figure 4. α-ERD Peak amplitude and latency for unisensory stimulation.**

**Supplementary Figure 5. Comparable Increases in Induced Theta-Band Activity Across Groups in Response to Different Sensory Modalities.** (**A**) Averaged topographical distribution of induced theta activity (4–7 Hz) in response to visual (left), auditory (center) and audiovisual (right) stimulation along with the corresponding time-frequency map for a cluster of occipital channels in response to the visual stimulation for NA (top row), AU (middle row), and SIB (bottom row). (**B**) Induced theta activity averaged over a cluster of centro-frontal channels (see corresponding topomaps) for NA (green), AU (orange), and SIB (purple) in response to the visual (left), auditory (center) and audiovisual (right) stimulation The shaded areas in the plots represent the standard error of the mean (SEM) in the corresponding colors.

**Supplementary Figure 6. wPLI between parieto-occipital and temporal cortex.** (**A**) Time-frequency representation of the weighted-phase lag index (wPLI) between a cluster of parieto-occipital channels and a cluster of temporal channels in response to a visual stimulation (left), auditory stimulation (center) and audiovisual (right) for the NA group (top row), AU group (middle line) and SIB group (bottom line). (**B**) wPLI averaged for the theta band (4-7Hz) for NA (green), AU (orange), and SIB (purple) for the corresponding sensory stimuli.

**Supplementary Figure 7. Correlation between wPLI and ERPs components and Reaction times.**
